# Gold Nanoparticle-Decorated Catalytic Micromotor-Based Aptassay for Rapid Electrochemical Label-Free Amyloid-β42 Oligomer Determination in Clinical Samples from Alzheimer’s Patients

**DOI:** 10.1021/acs.analchem.3c05665

**Published:** 2024-03-29

**Authors:** Álvaro Gallo-Orive, María Moreno-Guzmán, Marta Sanchez-Paniagua, Ana Montero-Calle, Rodrigo Barderas, Alberto Escarpa

**Affiliations:** † Department of Analytical Chemistry, Physical Chemistry and Chemical Engineering, University of Alcalá, Ctra. Madrid-Barcelona, Km. 33.600, 28802 Alcalá de Henares, Madrid, Spain; ‡ Department of Chemistry in Pharmaceutical Sciences, Faculty of Pharmacy, Complutense University of Madrid, Plaza Ramón y Cajal s/n, 28040 Moncloa-Aravaca, Madrid, Spain; § Chronic Disease Programme, UFIEC, Carlos III Health Institute, 28220 Majadahonda, Madrid, Spain; ∥ Chemical Research Institute “Andrés M. Del Rio”, University of Alcalá, 28802 Alcalá de Henares, Madrid, Spain

## Abstract

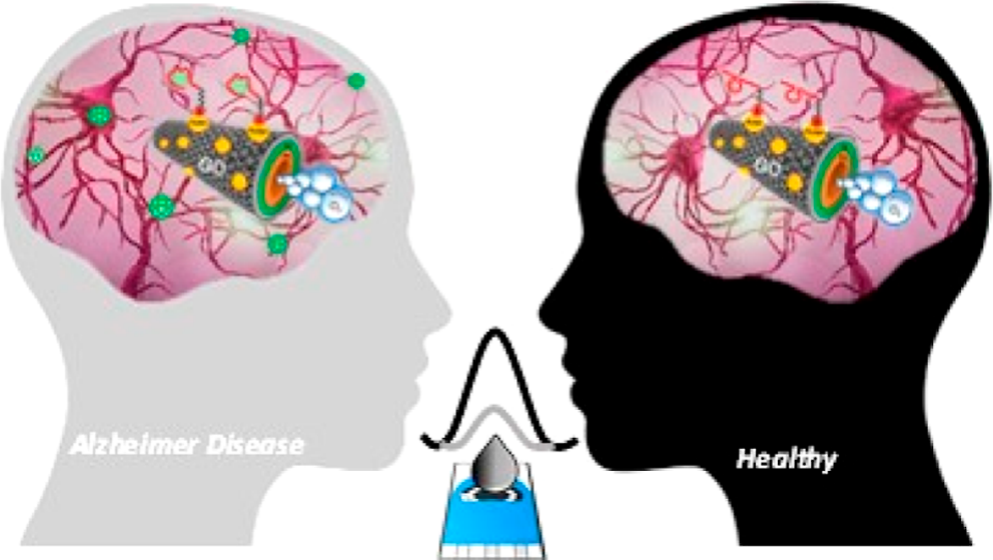

Micromotor (MM) technology offers a valuable and smart on-the-move biosensing microscale approach in clinical settings where sample availability is scarce in the case of Alzheimer’s disease (AD). Soluble amyloid-β protein oligomers (AβO) (mainly AβO_42_) that circulate in biological fluids have been recognized as a molecular biomarker and therapeutic target of AD due to their high toxicity, and they are correlated much more strongly with AD compared to the insoluble Aβ monomers. A graphene oxide (GO)–gold nanoparticles (AuNPs)/nickel (Ni)/platinum nanoparticles (PtNPs) micromotors (MM_GO–AuNPs_)-based electrochemical label-free aptassay is proposed for sensitive, accurate, and rapid determination of AβO_42_ in complex clinical samples such as brain tissue, cerebrospinal fluid (CSF), and plasma from AD patients. An approach that implies the in situ formation of AuNPs on the GO external layer of tubular MM in only one step during MM electrosynthesis was performed (MM_GO–AuNPs_). The AβO_42_ specific thiolated-aptamer (Apt_AβO_42__) was immobilized in the MM_GO–AuNPs_ via Au–S interaction, allowing for the selective recognition of the AβO_42_ (MM_GO–AuNPs_–Apt_AβO_42__–AβO_42_). AuNPs were smartly used not only to covalently bind a specific thiolated-aptamer for the design of a label-free electrochemical aptassay but also to improve the final MM propulsion performance due to their catalytic activity (approximately 2.0× speed). This on-the-move bioplatform provided a fast (5 min), selective, precise (RSD < 8%), and accurate quantification of AβO_42_ (recoveries 94–102%) with excellent sensitivity (LOD = 0.10 pg mL^–1^) and wide linear range (0.5–500 pg mL^–1^) in ultralow volumes of the clinical sample of AD patients (5 μL), without any dilution. Remarkably, our MM-based bioplatform demonstrated the competitiveness for the determination of AβO_42_ in the target samples against the dot blot analysis, which requires more than 14 h to provide qualitative results only. It is also important to highlight its applicability to the potential analysis of liquid biopsies as plasma and CSF samples, improving the reliability of the diagnosis given the heterogeneity and temporal complexity of neurodegenerative diseases. The excellent results obtained demonstrate the analytical potency of our approach as a future tool for clinical/POCT (Point-of-care testing) routine scenarios.

## Introduction

Catalytic tubular micromotors (MM) pioneered by Mei and Schmidt and Wang’s groups^1,2^ are usually made from the external sensing layer, magnetic layer for guidance, and catalytic layer for self-propulsion, usually toward oxygen bubbles ejection due to the decomposition of hydrogen peroxide on platinum-based catalysts. The chemically bubble-propulsion capability of MM produces an autonomous movement that induces efficient fluid mixing, accelerating chemical operations and, consequently, an ultrasensitive detection in short times in microscale environments.^3−6^

The use of Au on the MM catalytic layer to improve catalytic activity or movement in different media has been reported.^7−10^ However, few authors have designed MM-based sensing platforms in which gold is used as a functionalization support. Wang’s group describes the use of self-propelled micromachines that are functionalized with nucleic acid. These MM were fabricated using a custom photolithography process, where they were sputtered with a gold layer and modified with a binary self-assembled monolayer (SAM) formed by a specific thiolate capture probe and a short-chain 6-mercapto-1-hexanol.^11^ Another recent strategy described the use of poly(3,4-ethylenedioxythiophene) (PEDOT)–Au/peroxidase catalytic micromotors. Micromotors were synthesized by simplified template electrodeposition followed by covalent enzyme immobilization on a Au layer, which has also been synthesized by electrodeposition.^12^ In another study, aptamer-modified nanomotors [manganese oxide nanosheets-polyethyleneimine decorated with nickel/gold nanoparticles (MnO_2_–PEI/Ni/AuNPs–aptamer)] were used for the capture of human promyelocytic leukemia cells (HL-60) from a human serum sample.^13^ The AuNPs were synthesized in an additional electrodeposition step after Ni/MnO_2_–PEI formation. Finally, the concentration of separated cancer cells was determined, using other sensing platforms which do not involve the use of MM, an aptamer/gold nanoparticles poly(3,4-ethylene dioxythiophene)-modified glassy carbon electrode.

MM functionalization on board is a key step to obtain a tailored high-selective biomarker determination. In this context, aptamers are short synthetic single-stranded DNA or RNA oligonucleotides capable of specifically recognizing their target molecules. Generally, aptamers are developed in vitro via SELEX (systematic evolution of ligands by exponential enrichment), a method based on combinatorial chemistry that allows the selection of high-affinity oligonucleotide to the target molecule.^14−16^ A wide variety of target molecules, such as proteins, peptides, cells, hormones, or even entire cells can be recognized and bonded by aptamers.^17^ They also display several advantages over antibodies; the chemical synthesis of aptamer is less expensive to manufacture, has less variability between batches, and has very controlled postproduction modification with no loss activity. They are also more stable and robust to ambient conditions and smaller in size compared with antibodies.^18^ In addition, aptamers molecules can be easily labeled with different fluorescent molecules, granting the possibility of multiplexed analysis.^19−21^ Therefore, aptamers are promising biomolecules for the development of specific, robust, and cheaper biosensing strategies with applications in biomedicine. In this context, the aptamers-based MM have exhibited excellent analytical performance in the determination of protein biomarkers in neonatal sepsis diagnosis, revealing the high potency use of these biorecognition elements on board MM technology, especially in scenarios where sample availability is hard to obtain such dealing with neonatal patients.^22−24^

On the other hand, Alzheimer’s disease (AD) is the most common type of dementia. In 2020, it represented between 60 and 70% of all the dementia cases in the world, which means from 30 to 35 million people worldwide with 6–7 million new patients per year.^25,26^ It is expected that these numbers will increase through the years, over 66 million by 2030 and nearly 100 million by 2050^27,28^ due to population aging, especially in underdeveloped countries, as the advanced age means the biggest risk factor.^25^

The principal AD diagnosis is still the clinical assessment, more specifically, the clinical interview with the patient, and a cognitive and neuropsychological evaluation for the quantification of the pattern and severity of the cognitive deficit against age-related norms.^29,30^ The preclinical onset occurs silently years before the first symptoms appear.^31^ There is no cure for AD, but there are treatments that may change disease progression.^32^

AD is a multifactorial and biologically heterogeneous dementia^33^ that is caused by chronic, progressive, and irreversible central nervous system degeneration.^31,34^ This degeneration has been confirmed by neuropathological studies which include main characteristics such as extracellular senile plaque deposition, intracellular accumulation of neurofibrillary tangles, and neuronal degeneration loss.^34^ The disease produces the aggregation of amyloid-β protein (Aβ),^31^ abnormal forms of Tau proteins; oxidative stress, chronic neuroinflammation, synapse dysfunction, and ultimately neuronal death.^26,35^ Aβ is a small 39–43 amino acid residue derived from amyloid precursor protein in the brain of the patients.^31^ For many years, scientists thought that Aβ-induced neurotoxicity in cell culture and in vivo was associated with insoluble Aβ.^36,37^ Among the different forms, the most prevalent are the peptides made up of 40 (Aβ_40_) and 42 (Aβ_42_) amino acid residues.^38^ Different studies also indicate that small and soluble structures of Aβ oligomers (AβO) could be circulating in biological fluids and being a good biomarker for AD.^39,40^ In addition, findings indicate that compared to Aβ monomers, AβO is more toxic and is correlated much more strongly with AD,^41,42^ and the most toxic soluble oligomers are formed by Aβ_42_ (AβO_42_).^43^ Furthermore, AβO is believed to trigger the phosphorylation of the microtubules, thereby impeding the signal transfer of the neuron and finally inducing neuronal damage. Therefore, AβO has been recognized as a responsible molecular biomarker and therapeutic target for AD.^44,45^

Different techniques have been reported to detect soluble AβO_42_ such as surface-based fluorescence intensity distribution analysis (sFIDA),^46^ fluorescence microscopy,^47^ electrochemical methods,^44,48,49^ electrochemiluminescence,^50^ enzyme-linked immunosorbent assay (ELISA),^51−53^ surface plasmon resonance,^54^ Raman spectroscopy,^55^ and mass spectrometry.^56^ However, these methods are usually time-consuming, costly, and highly complex and require sophisticated instrumentation. For these reasons, alternative methods have been explored to improve sensitivity, selectivity, and simplicity.^57^ Considering that clinical samples of AD patients are hardly available and the conceptual reasons given above regarding the pertinence in the use of MM as a low-based sample diagnosis, these on-the-move biosensing platforms become an attractive approach.

In this work, a catalytic tubular MM–AuNPs-based approach (MM_GO–AuNPs_) for AβO_42_ determination is proposed. AuNPs were smartly and simultaneously used to covalently bind a specific thiolated-aptamer to the outer layer via a S–Au bond for the design of a label-free electrochemical aptassay and to improve the final MM propulsion performance due to catalytic activity. Elegantly, AuNPs were membrane-template coelectrosynthesized in situ with the graphene oxide sensing layer of MM. The proposed MM_GO–AuNP_-based aptassay was developed for sensitive, reliable, and fast on-the-fly recognition of AβO_42_ in samples with high and representative clinical significance from hospital patients with AD such as brain tissue, cerebrospinal fluid (CSF), and human plasma.

To our knowledge, this is the first approach involving the in situ formation of gold nanoparticles on the GO external layer of tubular MM for aptamer immobilization and the use of MM-based on-the-fly aptassays for AβO_42_ determination.

## Experimental Section

### Electrosynthesis of Au Nanoparticles–Graphene–Nickel–Platinum Nanoparticles MM (MM_GO–AuNPs_)

MM synthesis follows a protocol based on electrodeposition above a polycarbonate membranes (PC) membrane. The S4-branched side of 5 μm-diameter conical pores of the PC membrane was treated with a sputtered thin gold film to perform as a working electrode. The system is based on a Teflon cell with aluminum as an electrical contact to the working electrode, with the membrane assembled in the center of the system. This synthesis was based on the electrodeposition of three specific functional layers: the outer layer of graphene oxide and AuNPs (GO–AuNPs) for immobilizing the aptamer, nickel as the intermediate layer for magnetic guidance, and platinum nanoparticles (PtNPs) as the inner layer for catalytic propulsion.

First, the outer layer based on carbon compounds was synthesized by the reduction of a solution of HAuCl_4_ 0.25% (m/v) and GO 0.5 mg mL^–1^, H_2_SO_4_ 0.1 M, and Na_2_SO_4_ 0.5 M previously dispersed in a bath ultrasonication during 30 min and tip sonication for 4 min at 50% amplitude, followed by cyclic voltammetry through 10 cycles (+0.3 to −1.5 vs Ag/AgCl (3 M KCl), at 50 mV s^–1^). Second, the nickel tube layer was plated inside the GO–AuNPs layer by the galvanostatic method. To generate nucleation spots, 10 pulses of −20 mA are applied for 0.1 s, followed by a constant current of −6 mA for 300 s to grow the nickel layer. Third, the PtNP inner layer was deposited by amperometry at −0.4 V for 750 s from an aqueous solution containing 4 mM H_2_PtCl_6_ in 0.5 M boric acid.

Once the MM grew and finalized the depositions of the four materials, the sputtered gold layer membrane was gently hand-polished with a 1 μm alumina slurry. After this, the membrane was dissolved in methylene chloride for 30 min to completely release the microtubes. The washing procedure was performed by making use of a magnet-holding block, thanks to the Ni magnetic layer of the MM which allowed the easy elimination of the supernatant. Afterward, successive washes of MM with isopropanol (10 min, three times), ethanol (5 min, twice), and water (5 min, once) were used to get a neutral medium. All MM were stored in ultrapure water at 4 °C when not in use. The template preparation method resulted in reproducible thousands of MM_GO–AuNPs_ with similar size and shape using a single membrane.

### MM_GO–AuNPs_–Apt_AβO_42__–AβO_42_ Aptassay

Preparation of aptamer-modified MM (MM_GO–AuNPs_–Apt_AβO_42__) as a previous reagent to perform the in situ aptassay was accomplished by the addition of 10 μL of a 10 μM specific thiolated AβO_42_ aptamer (see Figure S1) solution to 25 μL of MM_GO–AuNPs_ into a test tube and incubated with a mechanical stirring incubation for 12 h at 25 °C in HEPES. Aptamers not bound onto the MM_GO–AuNPs_ were eliminated by twice washing with HEPES and washing with PBS.

In a second step, to ensure that the AβO_42_ binds only to the immobilized aptamer and not physically adsorbed to the MM_GO–AuNP_ surface producing a decrease in sensitivity,^58^ a 5% of bovine serum albumin (BSA) was used. This mixture was incubated under the same conditions as aptamer for 1 h. Then, it was washed 3 times with PBS to remove BSA not bound.

As the third step and to perform the AβO_42_ on-the-fly aptassay, a mix solution (5 μL of total volume) that contained the AβO_42_ sample, without dilution or the standard dissolved in PBS, and H_2_O_2_ (2%) were added. After 5 min of self-propelled motion of MM to recognize the AβO_42_, the solution was washed again 3 times with PBS and resuspended in 50 μL of Fe(CN)_6_^3–/4–^ (5 mM each; KCl 0.1 M; PBS 0.01 M) redox probe solution.

Finally, electrochemical measurements for AβO_42_ detection were performed by square wave voltammetry (SWV). When increasing the oligomer concentration, decrease the cathodic current due to the greater hindrance of Fe (CN)_6_^3–/4–^ to access the electrode (inner sphere model). The SWV signals were fitted to the following four-parameter logistic equation using the software SigmaPlot 10.
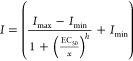
In this equation, and specifically for our assay, *I* is the cathodic current, *I*_max_ and *I*_min_ are the maximum and minimum current values of the calibration graph; the EC_50_ value is the analyte concentration corresponding to 50% of the maximum signal; and *h* is the hill slope.

On the other hand, detection limit (LOD) and quantification limit (LOQ) were calculated as 3*S*_0.5_/*m* and 10*S*_0.5_/*m*, respectively, where *S* is the standard deviation (*n* = 10) obtained during the measurement of the SWV from the lowest AβO_42_ concentration used in the calibration (0.5 pg mL^–1^) and *m* is the slope of the linear calibration plot.

## Results and Discussion

### Electrosynthesis and Characterization of MM_GO–AuNPs_ for AβO_42_ Determination

**Figure 1:**
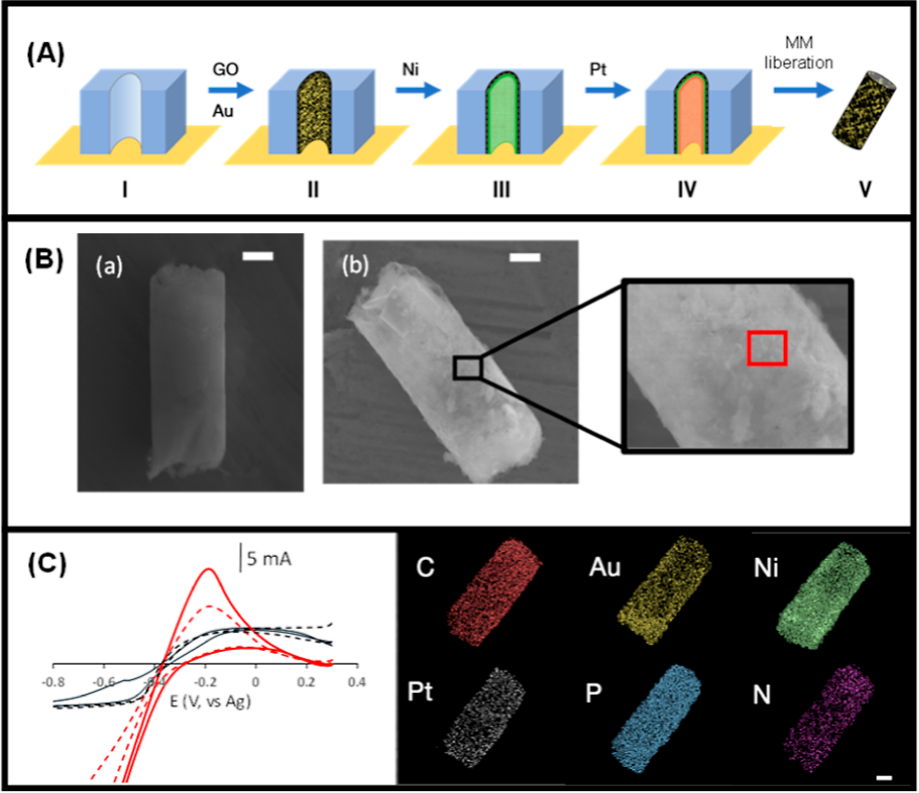
Schematics of the preparation of MM_GO–AuNPs_ (A), SEM images of MM_GO_ and MM_GO–AuNPs_ (B), and first (---) and last after 10 cycles (—) of the cyclic voltammetry of the outer layer electrosynthesis of MM_GO_ (black) and MM_GO–AuNPs_ (red) and EDX analysis of MM_GO–AuNPs_–Apt_AβO_42__ (C). Scale bar (B,C): 2 μm.

Figure 1 shows a schematic of the template-based electrosynthesis of MM_GO–AuNPs_ (A) and the characterization of MM_GO–AuNPs_–Apt_AβO_42__ using scanning electronic microscopy (SEM) (B) and X-ray spectroscopy analysis (EDX) (C) to demonstrate the successful electrosynthesis of MM_GO–AuNPs_ and aptamer covalent functionalization. The tubular catalytic MM were electrosynthesized by concentric layers with precise functions (Figure 1A): bare membrane (I); graphene oxide decorated with AuNPs outer layer (GO–AuNPs), as a functionalized support for the specific aptamer immobilization (II); a Ni intermediate layer for magnetic guidance and assistance in the washing stages of the aptassay (III); and an internal PtNPs catalytic layer for the generation of oxygen bubble-mediated propulsion in the presence of H_2_O_2_ fuel (IV); and removal of the template and MM liberation ready to be functionalized (V). SEM images of MM_GO–AuNPs_–Apt_AβO_42__ revealed a well-defined structural morphology based on a tubular shape with dimensions of 5 μm in width and 10 μm of length. Please note that MM without AuNPs (MM_GO_), (a) the layer is smoother while the MM_GO–AuNPs_ (b) have a less regular surface presenting those agglomerates of approximately 300 nm (shown in red square) that represent the AuNPs (Figure 1B). Figure 1C (left) shows the first and last cyclic voltammetry (CV) cycles for the electrosynthesis of the outer layers of MM_GO_ and MM_GO–AuNPs_. Differences in the CVs were observed between the outer layer of the MM_GO_ (black color) and MM_GO–AuNPs_ (red color) where only in MM_GO–AuNPs_, a clear oxidation peak (at −0.2 V/Ag), which increases with the number of cycles, was observed, which indicates the formation of Au NPs (for full CVs see Figure S2). EDX mapping confirmed the elemental composition of the MM homogeneously distributed (C and Au as the sensing layer, Ni as the magnetic layer, and Pt as the catalytic layer), demonstrating the efficiency of the MM electrosynthesis. Further, the EDX analysis indicates the existence of the phosphorus and nitrogen content, confirming the presence of aptamer on the MM surface (Figure 1C, right panel).

### MM_GO–AuNPs_-Based Aptassay Strategy for AβO_42_ Determination

**Figure 2:**
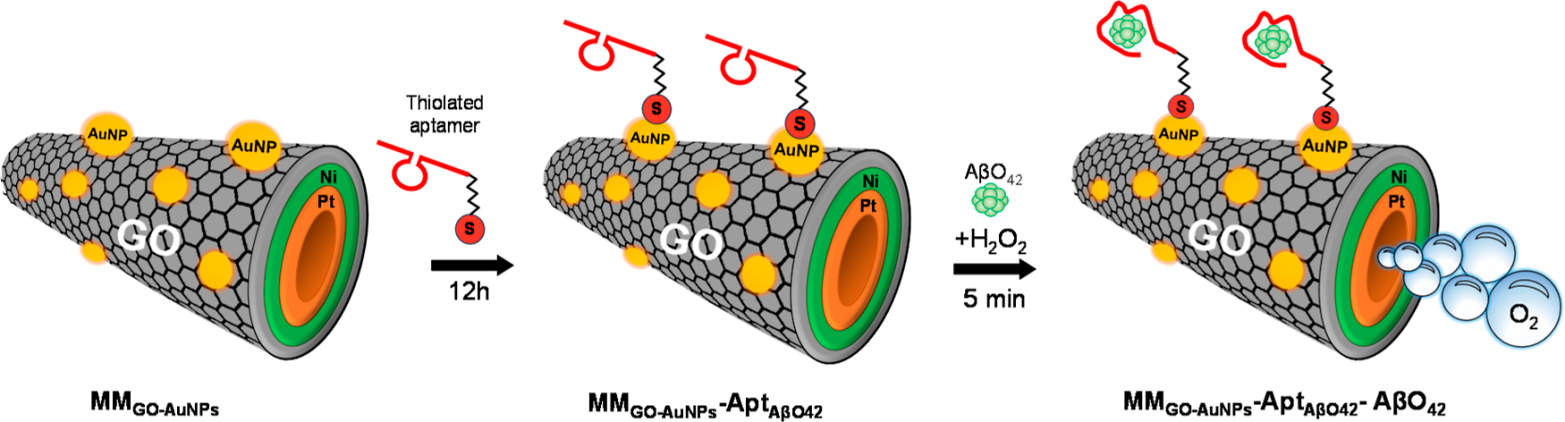
Schematics of the preparation of MM_GO–AuNPs_–Apt_AβO_42__–AβO_42_: MM_GO/AuNPs_ functionalization with the specific aptamer and on-the-fly aptassay are used for AβO_42_ determination.

Figure 2 illustrates the principle of on-the-fly aptassay based on the binding of an AβO_42_ by specific thiolated-aptamer covalently linked to the outer layer of MM_GO–AuNPs_ via a S–Au bond (MM_GO–AuNPs_–Apt_AβO_42__–AβO_42_) for its label-free electrochemical detection.

**Figure 3:**
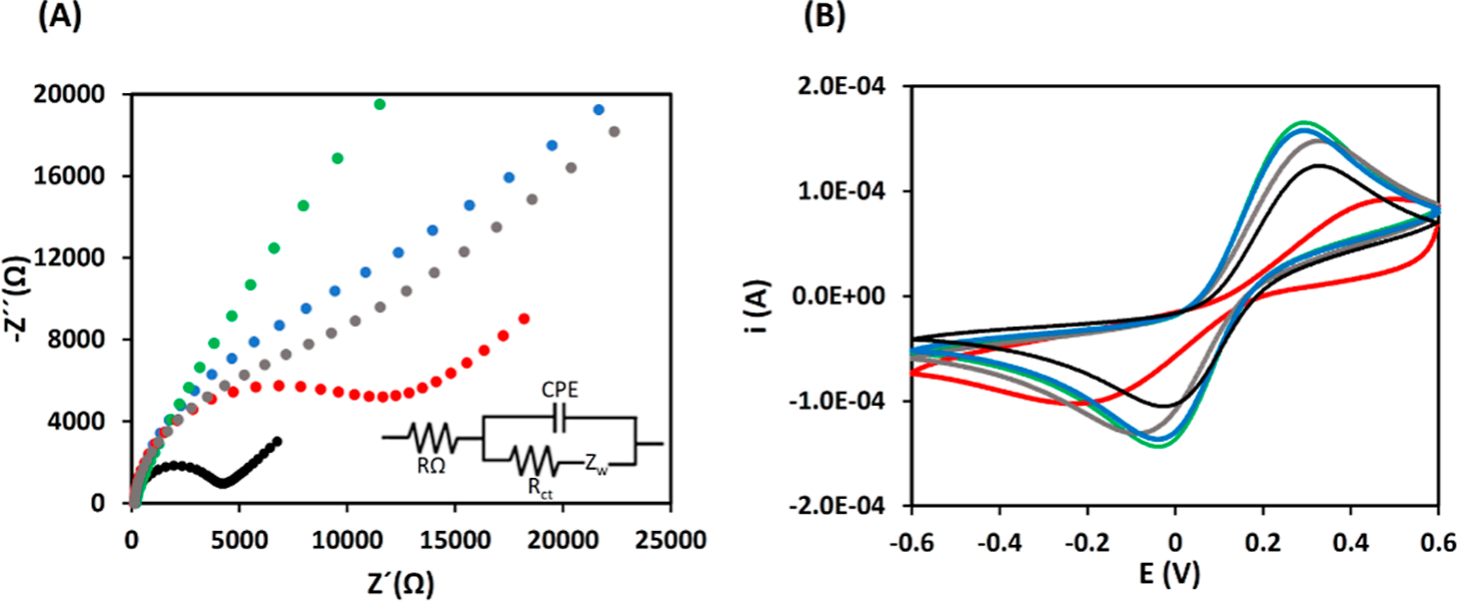
Nyquist plots (A) (inlet: equivalent electrical circuit diagrams for impedance plots) and cyclic voltammograms (B) of 5 mM Ru(NH_3_)_6_^3+/2+^ in PBS 0.01 M. SPCE (black), MM_GO–AuNPs_–SPCE (blue), MM_GO–AuNPs_–Apt_AβO_42__–SPCE (green), MM_GO–AuNPs_–Apt_AβO_42__–AβO_42_–SPCE (red) and MM_GO–AuNPs_–AβO_42_–SPCE (gray).

The study of the interface properties of the electrode surface during the fabrication procedure was performed by electrochemical impedance spectroscopy (EIS). Figure 3A shows that in the bare screen-printed carbon electrode (SPCE) appears a semicircle in the Nyquist plot with a charge transfer resistance (*R*_ct_) of 4311 Ω (black color) (unmodified electrode, control a). If the electrode is modified with MM_GO–AuNPs_, the semicircle disappears due to the conductivity of the AuNPs (blue color). When the aptamer is immobilized on the MM surface, the semicircle does not appear as a consequence of the attraction between the negatively charged phosphate backbone of the single strand DNA (ssDNA) aptamer and positively charged of the redox probe (Ru(NH_3_)_6_^2+/3+^) (green color). The modification with the presence of AβO_42_ has risen to a large increase in the charge transfer resistance (*R*_ct_ = 14,600 Ω), implying that the aptamerAβO_42_ complex has been formed at the electrode surface hindering the access of Ru(NH_3_)_6_^2+/3+^ to the electrode (red color). This semicircle does not appear if the oligomer is added directly to an MM_GO–AuNPs_–SPCE (without aptamer, control b) (gray color), suggesting the AβO_42_ specific union only in the presence of aptamer.

CV measurements were also carried out to confirm the EIS results (Figure 3B). The redox current in bare SPCE shows an *I*_p_ of 0.090 mA and an Δ*E*_p_ of 314 mV (black color). The addition of MM_GO–AuNPs_ implies an increase in the *I*_p_ (0.140 mA) and a small reduction of the Δ*E*_p_ (306 mV) due to the conductivity properties of the MM material (blue color). After the immobilization of the aptamer, the voltammetry cyclic profile practically does not vary (*I*_p_ of 0.138 mA and Δ*E*_p_ to 300 mV) due to the attraction charges of the aptamer-redox probe (green color). In contrast, MM_GO–AuNPs_–Apt_AβO_42__–AβO_42_–SPCE exhibited a drastic decrease in peak current (*I*_p_ = 0.033 mA) and a higher separation between the two peak potentials (Δ*E*_p_ = 693 mV) showing an irreversible process which indicates that the oligomer was successfully binding by the aptamer hindering the access of the Ru (NH_3_)_6_^2+/3+^ probe (attached on the electrode surface, red color). Again, when an oligomer is added to MM_GO–AuNPs_-modified electrodes (gray color), the profile of the CV is like the one obtained with only MM, suggesting the specific recognition of AβO_42_ only in the presence of aptamer.

### Optimization of the MM-Based Aptassay

The main experimental variables affecting the preparation of the MM-based bioplatform were tested by EIS using 5 mM Ru(NH_3_)_6_^3+/2+^ as a redox probe to detect the *R*_ct_ signal caused by AβO_42_ binding to the aptamer receptor. Because the incubation of the aptamer (MM_GO–AuNPs_–Apt_AβO_42__–SPCE) did not influence *R*_ct_, the *R*_ct_ obtained after the biorecognition of the AβO_42_ oligomer was adopted as the selection criterion of the checked variable. Table 1 lists the tested ranges and values selected for all experimental variables assayed.

**Table 1:** Optimization of the Variables Involved in the MM-Based Aptassay

variable	tested range	selected value
HAuCl_4_, %	0.05–0.5	0.25
MM, μL (number)	5–50 (1000–10,000)	25 (5000)
aptamer volume, μL	1–25	10
[aptamer], μM	0.1–25	10
aptamer incubation time, h	1–18	12
fuel (H_2_O_2_), %	0.5–4	2
sample volume, μL	2–50	5
recognition time, min	2–25	5

Figure S3 shows the optimization of the variables involved in the formation of MM_GO–AuNPs_. As can be seen, an increase in the *R*_ct_ value is observed with the increase of HAuCl_4_ in the MM synthesis, obtaining the highest signal (*R*_ct_ = 20,300 Ω) with 0.25% of HAuCl_4_ (Figure S3A), demonstrating that the AβO_42_–Apt_AβO_42__ complex is greater when 0.25% of HAuCl_4_ is used to create the GO–AuNPs layer, where the specific thiolated-aptamer will be attached via the S–Au bond. As shown in Figure S3B, different volumes of MM attached to the electrode surface were evaluated between 5 and 50 μL (approximately 1000 and 10,000 micromotors, respectively). As the higher amount of MM produced a constant signal, they gave rise to aggregations, diminishing the active surface and lowering the effective navigation to capture the analyte. For this reason, the optimized amount of micromotors was chosen to have enough binding sites to form the highest amount of aptamer bind on the MM without such aggregation occurring in MM. A compromise situation between greater efficiency of oligomer union and higher blocking effect with the increase in the number of MM must be reached. An increase in the *R*_ct_ is observed up to 25 μL (*R*_ct_ = 23,200 Ω) due to the increase of binding sites for aptamer molecules. For a higher volume of micromotors, no change in *R*_ct_ occurs probably because the magnetic MM agglomeration does not produce a significant change in the active surface for aptamer bonded.

Figure S4 shows the optimization of the variables involving the aptamer immobilization and the on-the-fly aptassay using the initial conditions 10 μL of 10 μM of aptamer during 1 h of incubation, 20 μL of sample volume, and on-the fly interaction during 10 min with 2% of H_2_O_2_ as fuel. The influence of the aptamer amount bound to the MM surface for the correct bond of the oligomer is studied in terms of aptamer volume (1–25 μL) and aptamer concentration (0.1–25 μM). The bound of aptamer time (1–18 h) was also optimized. As observed in Figure S4A,B, 10 μL and 10 μM of aptamer, respectively, produced the highest *R*_ct_ value in the Nyquist plot (18,000 Ω), implying high aptamer–oligomer complex immobilized (MM_GO–AuNPs_–Apt_AβO_42__–AβO_42_–SPCE). Higher amounts of aptamer molecules show a decrease in *R*_ct_ (*R*_ct_ of 14,100 Ω for 25 μM), probably producing steric impediments. The optimum incubation time was 12 h (Figure S4C; this is not an inconvenience because it is a duration compatible with overnight incubation and its stability). The optimization of the variables involving the on-the-fly aptassay, sample volume and affinity reaction time (formation of the AβO_42_–Apt_AβO_42__ complex), was also studied (Figure S4D,E, respectively). The optimum conditions were 5 μL of the sample and 5 min. MM swimming (*R*_ct_ = 31,000 Ω) highlights the excellent characteristics of the aptassay in terms of low volume of the sample and short analysis time to be able to detect AβO_42_ with great sensitivity. The efficiency of the affinity on-the-fly interaction is highly influenced by the fuel concentration used. The highest signal (*R*_ct_ = 35,250 Ω) is observed with 2% H_2_O_2_ (Figure S4F). The descent observed for a high concentration of H_2_O_2_ (*R*_ct_ of 9500 Ω for 4%) can be explained by the lower probability of oligomer–aptamer interaction due to the high speed of the MM. If an excessively high fuel concentration is used, the recognition event becomes less efficient as the high velocity of the MM prevents sufficient time for an effective aptamer–oligomer interaction.

Another variable to consider in the aptassay is the possible addition of a blocking agent to improve the sensitivity. The presence of bovine serum albumin (BSA) produces an increase in sensitivity, probably due to avoiding the adsorption of oligomer molecules directly to the MM surface. 5% of BSA was selected as the optimum value, which is also the approximate average of albumin serum in human plasma.^22^

The self-propelled advantages of MM_GO–AuNPs_ were evaluated by comparison with other MM propulsion conditions such as external stirring or static conditions. In all cases, the cathodic current of SWV for 0 (Signal Blank, B), 0.5, and 10 pg mL^–1^ of AβO_42_ (Signal, S) was measured. The highest difference of S_10_/B ratio (signal MM_GO–AuNPs_–Apt_AβO_42__–AβO_42_/signal MM_GO–AuNPs_–Apt_AβO_42__ using 5 mM Fe(CN)_6_^3–/4–^ in 0.1 M KCl, PBS 0.01 M) was obtained for the MM approach (0.6) in comparison with static (0.8) and stirring (0.7). If the concentration of oligomer detected is very low, 0.5 pg mL^–1^, the *S*_0.5_/*B* ratio was 0.9 for the MM approach and approximately equal to 1 (no variation was observed) for static and stirring conditions. These results indicated the MM-induced mixing performance due to high speed and bubble trail (please note that a decrease of the MM_GO–AuNPs_–Apt_AβO_42__–AβO_42_ signal means improved specific interaction with AβO_42_). Consequently, an ultrasensitive detection in short times in microscale environments would be possible with self-propelled MM but go practically unnoticed with the other MM propulsion conditions, mainly at the lowest concentrations.

MM_GO–AuNPs_ were able to move during the whole on-the-fly assay time, showing their high propulsion capabilities even when the sample volume was low (5 μL) and in complex clinical samples (brain tissue, CSF, and plasma), allowing great efficiency of the assay (Video S1). If MM_GO_ and MM_GO–AuNPs_ are compared, MM_GO–AuNPs_ showed a higher speed (approximately 2.0×) in PBS with 2% of H_2_O_2_ due to the presence of AuNPs on their surface (120 ± 30 vs 229 ± 40 μm s^–1^). As expected, a decrease in the speed (130, 175, and 188 μm s^–1^) is noted when navigating in complex samples (brain tissue, CSF, and plasma); however, it does not hamper the motion or efficient swimming behavior of the micromotors, showing yet a remarkably high speed. It is worth mentioning that after the binding of the aptamer to the MM, a decrease in the speed of 10–15% was also observed in the samples studied. Again, the decrease in speed does not prevent the success of the MM biosensing of AβO_42_ on board.

### Analytical Performance of the MM-Based Aptassay

To obtain a highly sensitive label-free-based aptassay, the oligomer quantification study was performed using square wave voltamperometry in the presence of Fe (CN)_6_^3–^/^4–^. Analytical characteristics of the aptassay were studied in the optimized conditions.

**Figure 4:**
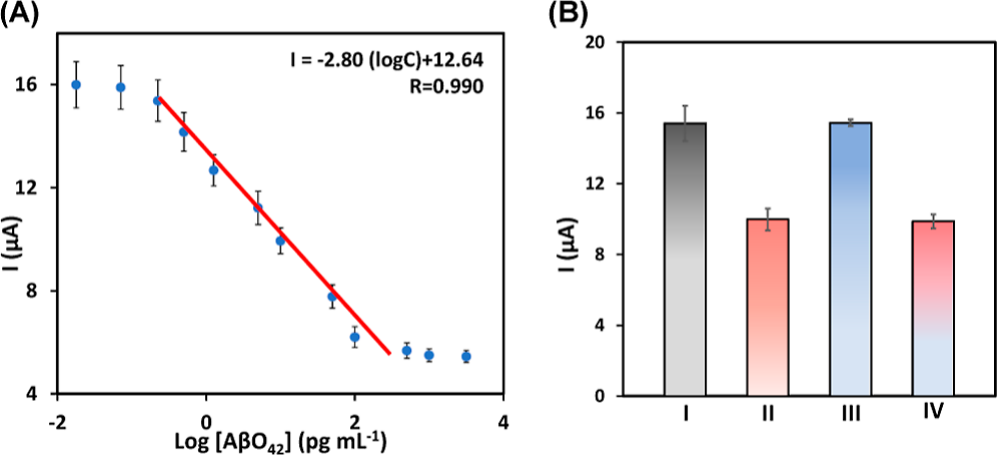
Sigmoidal calibration plot (A); interference study of the aptassay (10 pg mL^–1^ of Aβ_42_ and AβO_42_), the signals were recorded from analyzing MM_GO–AuNPs_–Apt_AβO_42__ (gray, I), MM_GO–AuNPs_–Apt_AβO_42__–AβO_42_ (red, II), MM_GO–AuNPs_–Apt_AβO_42__–Aβ_42_ (blue III), and MM_GO–AuNPs_–Apt_AβO_42__–AβO_42_/Aβ_42_ (degraded red–blue, IV) (B).

A linear relationship between intensity and logarithm of AβO_42_ concentrations was obtained (Figure 4A). Calibration performance exhibited a linear range of 0.5 to 500 pg mL^–1^ (*r* = 0.990), suitable for clinical practice as well as a very good sensitivity with LOD = 0.10 pg mL^–1^ and LOQ = 0.30 pg mL^–1^. The selectivity of the aptassay was tested in the presence of 10 pg mL^–1^ of amyloid beta peptide (Aβ_42_), as shown in Figure 4B. The response for MM_GO–AuNPs_–Apt_AβO_42__–Aβ_42_–SPCE (III) is like the one obtained with MM_GO–AuNPs_–Apt_AβO_42__–SPCE (I). Further, a mixture of Aβ_42_ and AβO_42_ (IV) shows a response like that of the MM_GO–AuNPs_–Apt_AβO_42__–AβO_42_–SPCE (II). These results confirm the selective union of the oligomer to the aptamer.

The precision was also evaluated by assaying different concentration levels of AβO_42_, minimum (0.5 pg mL^–1^), a value close to EC_50_ (12 pg mL^–1^), and maximum (500 pg mL^–1^) with values of RSD < 8% (*n* = 5). These results demonstrated the good intraassay repeatability (same day) and intermediate precision (different days) of the on-the-fly aptassay (Table 2).

**Table 2:** Precision Study of the MM-Based Aptassay

concentration assayed (pg mL–^1^)	intra-assay precision CV (%) (*n* = 5)	intermediate precision CV (%) (*n* = 5)
0.5	6	6
10	5	6
500	6	8

Due to the potential use of MM_GO–AuNPs_–Apt_AβO_42__ complexes as a POC, to simplify the entire procedure and, in turn, to reduce the final analysis times (only 5 min), the stability of the MM_GO–AuNPs_–Apt_AβO_42__ complexes to be used as stock “reagents” was studied. MM_GO–AuNPs_–Apt_AβO_42__ were prepared the same day at 10 μM as the aptamer loading concentration followed by a blocking step with BSA and stored at 4 °C in PBS 0.01 M. The aptassay remained inside the control limits placed at ± three times the standard deviation value calculated for the whole set of experiments, during the entire period checked (15 days). These results (not shown) demonstrate the excellent stability of the MM_GO–AuNPs_–Apt_AβO_42__ complexes.

### Sample Analysis from AD Patients

**Figure 5:**
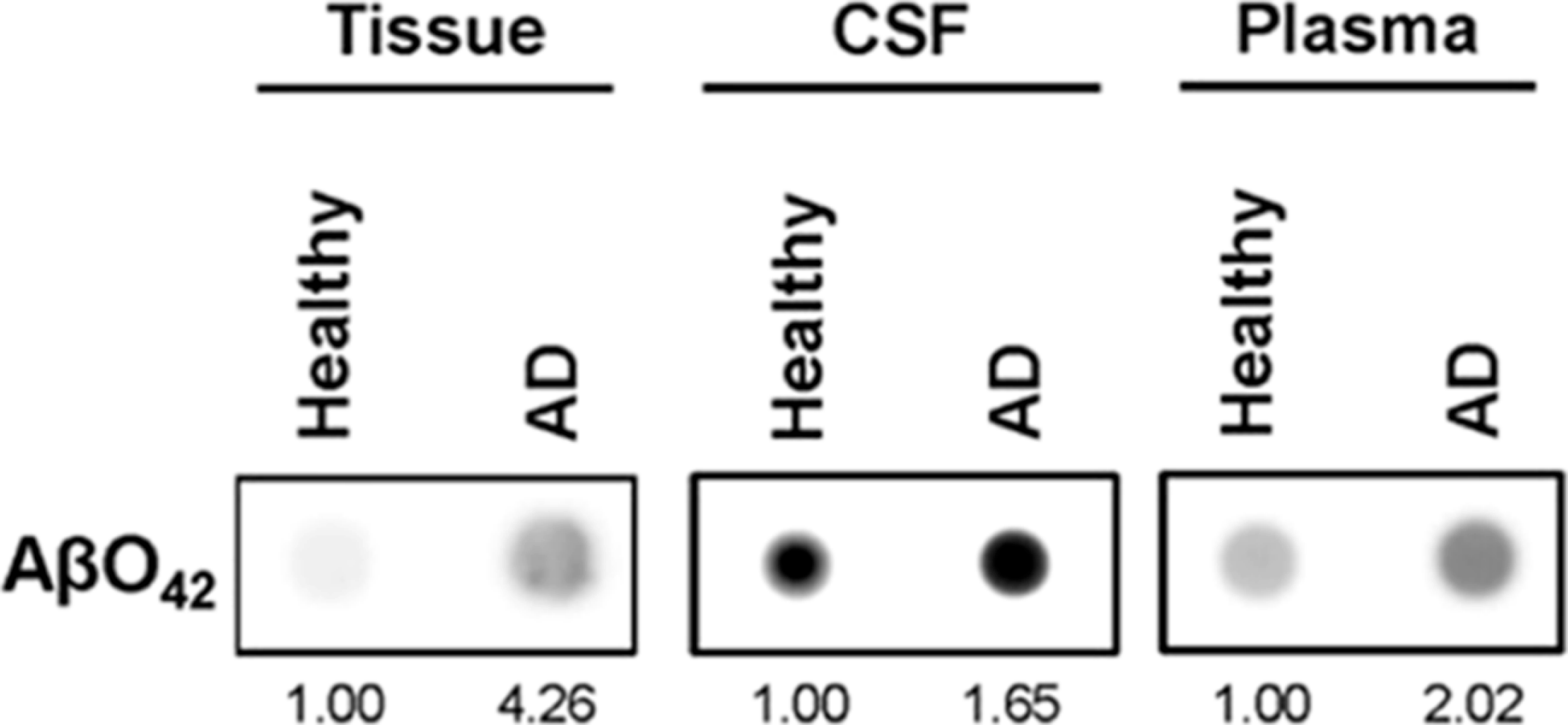
Qualitative detection of AβO_42_. Dot blot assessment of AβO_42_ in tissue (brain tissue protein extract), CSF, and plasma in representative samples from AD patients and controls (Table 3). Samples were blotted onto nitrocellulose membranes and probed with AβO_42_ antibody. Protein intensities were referred to as the intensity of AβO_42_ in the samples from the control individuals.

Table 3 lists the quantitative analysis of brain tissue, CSF, and plasma using the aptassay for AβO_42_ determination in both samples from healthy individuals, controls, (non- and spiked ones) and diagnosed AD patients. Also, an analysis of non and spiked commercial serum samples was carried out. The excellent quantitative recovery percentages obtained showed the accuracy of the developed aptassay for the determination of AβO_42_ during the analysis of the samples from commercial serum and healthy individuals. Then, more importantly, this aptassay was also tested by the demanding analysis of clinical samples from patients with confirmed Alzheimer’s diagnosis, where samples are very difficult to obtain and the volume of samples available is extremely scarce. An increase in the AβO_42_ levels was observed, without exception, in all types of AD-diagnosed clinical samples in comparison to those obtained in healthy individuals. The aptassay was also evaluated by dot blot assessment (Figure 5), confirming higher AβO_42_ oligomer levels in all samples from AD patients in comparison with healthy individuals as the control, in qualitative agreement with results obtained in the MM-based aptassay. Indeed, the correlation was obtained in the data comparing the protein expression results from dot-blot (higher expression in AD patients in comparison to control individuals) and the AβO_42_ aptassay. However, it is worth mentioning that quantification of AβO_42_ in brain tissue, CSF, and plasma extracts was only accomplished with the MM-based approach, with dot blot analyses showing qualitative results only.

**Table 3:** Analysis of Brain Tissue, CSF, and Plasma Using the MM-Based Aptassay for AβO_42_ Determination in Samples From Healthy Individuals and AD Diagnosed Patients^a^

	healthy individuals				AD patients
sample	AβO_42 determined_ (pg mL^–1^)	AβO_42 added_ (pg mL^–1^)	AβO_42 found_ (pg mL^–1^)	recovery (%)	AβO_42_ (pg mL^–1^)
serum		50	48 ± 3	96 ± 5	
brain tissue	4.0 ± 0.6	50	53 ± 4	98 ± 6	360 ± 60
CSF	0.8 ± 0.1	50	52 ± 2	102 ± 4	200 ± 10
plasma	5.8 ± 1.3	50	53 ± 3	95 ± 4	96 ± 8

a Values are given as mean value ±SD (*n* = 5).

Comparison with the literature is a difficult matter due to the inherent complexity of knowing reliable quantitative levels in the different samples studied. This comparison will be discussed below in two differentiated parts: first, the levels of AβO_42_ found in our approach will be compared with those reported in the literature in this type of clinical samples, and then, the analytical characteristics of the MM-based aptassay will be compared in comparison with other approaches that use the same detection principle. According to the literature, the concentration range of the biomarker AβO in the blood and CSF of AD patients is reported to be between 5.5 and 200 pM.^59−61^ Similar orders of magnitude were observed in brain tissue.^62^ Consequently, the MM-based electrochemical aptassay detection capabilities (0.02 pM) will allow differentiation of healthy and AD patients. Furthermore, different studies reveal significant differences in AβO concentrations for AD patients and control groups in different samples. Savage et al. show a significant 3- to 5-fold increase in Aβ oligomers in CSF compared with comparably aged controls.^63^

Other studies showed levels of AβO in CSF from AD patients to be 30-fold higher than those from nondemented individuals.^64^ Yang et al. revealed an AβO concentration in brain tissue for AD 50-fold higher than those of the age-matched controls.^65^ These differences could be due to the correlation in the Aβ oligomer level and the different stages of AD.^63,66^ In our study, a significant increase in the AβO_42_ concentration, in all actual samples from AD patients compared with controls, was obtained (16-fold elevated in plasma, 90-fold increase in brain tissue, and 250-fold higher in CSF). It is worth noting the higher AβO_42_ concentration was observed in our approach in brain tissue and CSF in AD patients compared with those obtained in plasma, which has also been previously observed in other studies.^62,67^

Foremost, this is the first MM-based aptassay for the AβO_42_ determination. For this reason, this approach is compared with other published articles involving label-free electrochemical assays for Aβ oligomer detection which use different immobilization systems such as AuNPs, monolayers, polymers, or composites, among others (Table S1). In most of the reports, the determination of the oligomer is carried out in a cell conditioned medium, artificial, or enriched biological fluids,^68,69^ and only one work analyzed the real plasma samples of AD patients.^70^ The present work provides an analysis of clinically relevant complex samples of AD patients such as brain tissue, CSF, and plasma. In this context, our approach allows obtaining similar sensitivity found in the literature (LOD = 0.02 pM) and the determination of AβO_42_ in diagnosed samples, which are reported here for the first-time giving value to MM technology for diagnostics, highlighting the very low sample volume used, the smallest one reported (5 μL)^70−73^ as well as the fastest assay (5 min) due to the inherent properties of self-propelled tubular micromotors. All this leads to placing our MM-based aptassay as a competitive biosensing approach for the determination of AβO_42_ as a relevant biomarker of AD exhibiting a high sensitivity and a linear working range that allows performing the sample analysis without dilution.

## Conclusions

A novel MM_GO–AuNPs_-based electrochemical label-free aptassay was successfully applied for the sensitive and selective determination of AβO_42_ in clinical complex samples of brain tissue, CSF, and plasma of AD patients. The in situ AuNP decoration/coelectrosynthesis of the MM_GO_ sensing layer has given the option of being able to covalently bind the recognition biomolecule in addition to improving their swimming speed. This on-the-fly aptassay exhibited excellent capabilities of sensitivity (LOD = 0.10 pg mL^–1^), reliability, and fast determination of AβO_42_. Just 5 min moving the MM through low sample volumes (only 5 μL) without prior preparation is sufficient to detect the oligomer in clinical samples. Even more importantly, the linear range covered the clinical levels, allowing the direct determination without any dilution, simplifying the analysis. The performance of the approach exhibits agreement concerning the qualitative analysis obtained by dot-blot. Remarkably, the application reported here demonstrates the competitiveness of the MM-based methodology developed for the determination of AβO_42_ in brain tissue protein extracts, CSF, and plasma against the dot blot analysis, which requires 5.0–15.0 μg of the protein content and more than 14 h to provide qualitative results only. It is also important to highlight the applicability of the on-the-move bioplatform to the analysis of different complex samples, including liquid biopsies as plasma and CSF samples, therefore improving the reliability in the diagnosis given the heterogeneity and temporal complexity of neurodegenerative diseases. Additionally, our approach is the first one using MM to measure actual AD patients’ samples previously confirmed based on international consensus criteria according to established neuropathological methodologies and classification at the CIEN foundation.^74,75^

In summary, this label-free aptassay becomes highly competitive not only with previous Aβ oligomers electrochemical bioassays but with traditional routine clinical methods becoming a realistic promise as a future point of care in AD disease. Furthermore, this proof-of-concept aptamer functionalized MM-based label-free strategy has the potential for the development of new and competitive approaches for further analyses and, potentially, patient management of diverse types of dementia diseases.

Although the potential of MM technology in the biosensing of relevant biomarkers in miscellaneous environments has been demonstrated, the implementation of this technology in the field of clinical diagnosis is still in its infancy, and at least two important challenges must be overcome. First, it promotes effective collaboration with hospitals and entities interested in transferring the technology. Second, it will be required that healthcare personnel expand their knowledge about this technology and how to implement it in their clinical practice, particularly in diagnoses with low availability of clinical samples, an area in which MM have enormous potential.

## Supporting Information Available

Experimental section and optimization of the variables involving aptassay of AβO_42_ (PDF)
Movement of the MM_GO–AuNPs_ during the on-the-fly aptassay time in different samples (MP4)

## Abbreviations

Aβ: amyloid-β protein
AβO: amyloid-β protein soluble oligomers
AβO_42_: amyloid-β42 oligomer
AD: Alzheimer’s disease
Apt: aptamer
AuNPs: gold nanoparticles
CSF: cerebrospinal fluid
CV: cyclic voltammetry
EIS: electrochemical impedance spectroscopy
GO: graphene oxide
LOD: detection limit
LOQ: quantification limit
Ni: nickel
PC: polycarbonate membranes
PtNPs: platinum nanoparticles
MM: micromotors
*R*_ct_: charge transfer resistance
SPCE: screen-printed carbon electrodes
SWV: square wave voltammetry
