# Supplementary material for: Gold Nanoparticle-Decorated Catalytic Micromotor-Based Aptassay for Rapid Electrochemical Label-Free Amyloid-β42 Oligomer Determination in Clinical Samples from Alzheimer’s Patients
Source: Anal Chem. 2024 Mar 29;96(14):5509–18. doi: 10.1021/acs.analchem.3c05665 (PMC11007680; doi:10.1021/acs.analchem.3c05665)
Supplement: Supplementary file 1 — ac3c05665_si_001.pdf [file ac3c05665_si_001.pdf]

SUPPORTING INFORMATION

GOLD NANOPARTICLE-DECORATED CATALYTIC MICROMOTORS-BASED APTASSAY FOR RAPID ELECTROCHEMICAL LABEL-FREE AMYLOID-β42 OLIGOMER DETERMINATION IN CLINICAL SAMPLES FROM ALZHEIMER'S PATIENTS

Álvaro Gallo-Orive[1,2], María Moreno-Guzmán[2*], Marta Sanchez-Paniagua[2], Ana Montero-Calle[3], Rodrigo Barderas[3], Alberto Escarpa[1,4*]

1 Department of Analytical Chemistry, Physical Chemistry and Chemical Engineering, University of Alcalá, Ctra. Madrid-Barcelona, Km. 33.600, 28802 Alcalá de Henares, Madrid, Spain

2 Department of Chemistry in Pharmaceutical Sciences, Faculty of Pharmacy, Complutense University of Madrid, Plaza Ramón y Cajal s/n, 28040 Madrid, Spain.

3 Chronic Disease Programme, UFIEC, Carlos III Health Institute, Majadahonda, Madrid 28220, Spain.

4 Chemical Research Institute "Andrés M. Del Rio", University of Alcalá, Madrid, Spain.

Email corresponding author: alberto.escarpa@uah.es, co-corresponding author: marimore@ucm.es

Table of content

EXPERIMENTAL

Reagents......S3

Apparatus......S3

Samples, protein extracts, and dot-blot......S4

Electrochemical measurements......S4

FIGURES

Figure S1......S5

Figure S2......S5

Figure S3......S6

Figure S4......S6

TABLES

Table S1......S7

REFERENCES

References......S8

Reagents

Human $A\beta O_{42}$ was purchased from Biosensis (Redfern, Australia), and human $A\beta_{42}$ from BioLegend (San Diego, California). The sequence of $A\beta O_{42}$ aptamer was 5'-SH-C6-TTTTTTTTTTGCCTGTGGTGTTGGGGCGGGTGCG-3' and was synthesized by Aptus Biotech (Madrid, Spain). Mfold Web Server was used to predict the secondary structure of the aptamer[1]. For this analysis, 25ºC and the saline concentration of the aqueous buffer employed were considered. The aptamer predicted to have two possible stable secondary structures (ΔG = -1.23 Kcal/mol and ΔG =-0.59 Kcal/mol respectively) (**see Figure S1**).

The aptamer was reconstituted in 25mM of 4-(2-hydroxyethyl)-1-piperazineethanesulfonic acid (HEPES) from Proquinorte (Zamudio, Spain) pH 7.0-7.6. Dilution of $A\beta O_{42}$ and $A\beta_{42}$ were prepared in phosphate-buffered saline (PBS) solution pH 7.4 (0.1 M Na_2HPO_4 (99%), 2.7 mM KCl (99%)) from Scharlau (Madrid, Spain; 0.1 M NaH_2PO_4, 138 mM NaCl (99%) from Panreac (Madrid, Spain)).

Graphene oxide (GO) (4 mg mL^{-1} dispersion in H_2O), hydrogen peroxide (H_2O_2) (30% v/v), sulfuric acid (H_2SO_4), sodium sulfate (Na_2SO_4), hexachloroplatinic (IV) acid (H_2PtCl_6), nickel (II) sulfamate tetrahydrate ($H_4N_2NiO_6S_2$), nickel (II) chloride hexahydrate (Cl_2NiO_6 H_2O), gold trichloride hydrochloride ($HAuCl_4$), boric acid (99.5%), dichloromethane (CH_2Cl_2), isopropanol, ethanol were purchase from Sigma-Aldrich (Madrid, Spain). Bovine serum albumin (BSA) was purchased from Sigma-Aldrich (Madrid, Spain) and diluted in PBS. 5 µm-diameter conical pores polycarbonate membranes (PC) were purchased from Whatman (Maidstone, UK).

Potassium hexacyanoferrate (II) ($Fe(CN)_6^{4-}$), Potassium hexacyanoferrate(III) ($Fe(CN)_6^{3-}$), hexaammineruthenium (II) chloride ($Ru(NH_3)_6^{2+}$) and hexaammineruthenium (III) chloride ($Ru(NH_3)_6^{3+}$) were purchased from Sigma Aldrich (Madrid, Spain).

All standard solutions used were of analytical-grade reagents, and deionized water was obtained from a Millipore Milli-Q purification system (18.2 MΩ cm at 25 °C) and protected from light.

Apparatus

An electrochemical station µ-Autolab PGSTAT100 from Metrohm (Herisau, Switzerland) performed with NOVA was used for template-assisted electrochemical deposition of $MM_{GO\text{-}AuNPs}$. All voltammetry and impedance measurements were performed, at room temperature, on an electrochemical station PGSTAT-204 (Autolab, Utrecht, Holland). The screen-printed carbon electrodes (SPCE) used were the 110 DropSens (DRP-110 SPCE) from Metrohm, which is optimum for 50 µL drop and uses a conventional three-electrode system comprising of carbon working and auxiliary electrodes and silver as reference electrode.

An inverted optical microscope (Nikon Eclipse 80i upright micro-scope), a Hamamatsu digital camera C11440, and NIS Elements AR 3.2 software were used for capturing images and movies of the micromotors. Advanced VortexMixer-ZX3 from VWR and Thermosaker TS-100 C from Biosan were used for incubation stages. Magnetic block DynaMag-2 obtained from ThermoFisher was used for the handling of magnetic $MM_{GO\text{-}AuNPs}$ A tip sonication VCX130 (Sonics, Newtown, USA), and a bath ultrasonication 3000683 Ultrasons Selecta (Barcelona, Spain) was used for dispersed solution of GO.

Scanning electron microscopy (SEM) images were obtained with a JEOL JSM 6335F instrument and X-ray analysis was performed through an EDX detector attached to a SEM instrument.

Samples, protein extracts, and dot-blot

The synthetic human serum from human male blood type AB was obtained from Sigma-Aldrich (Madrid, Spain). Plasma, brain tissue, and cerebrospinal fluid samples from healthy controls and from patients diagnosed with Alzheimer's disease (AD patients) were stored at -80 ºC until their use. The samples were provided by the CIEN Foundation's Tissue Bank (BT-CIEN) in compliance with ethical issues and brain bank's guidelines. Written informed consent was obtained from all participants. The Institutional Ethical Review Board of the Spanish Research Center for Neurological Diseases Foundation (CIEN), the Instituto de Salud Carlos III, and the University of Alcalá de Henares approved this study on the analysis of biomarkers of Alzheimer's disease (CEID2021/4/108).

Brain tissue protein extracts were obtained following established protocols[74,75]. Qualitative analysis of $A\beta O_{42}$ in brain tissue protein extracts, CSF and plasma of AD and control individuals was performed by means of dot blot analysis. In brief, 2 µg of plasma, 15 µg of brain tissue protein extract, and 5 µg of CSF in 100 µL PBS were dot blotted onto nitrocellulose membranes using the Bio-Dot 96-Well Microfiltration (Bio-Rad). Then, membranes were processed using the A11 antibody for the detection of oligomers as previously described[2]. Signal was developed with the ECL Pico Plus chemiluminescent reagent (Thermo Fisher Scientific) and detected using an Amersham Imager 680 (GE Healthcare). Protein band intensities were quantified using ImageJ Software.

Electrochemical measurements

Electrochemical Impedance Spectroscopy (EIS) and cyclic voltammetry (CV) were employed to study the interface properties of the modified electrode surface during the fabrication procedure of the aptassay.

CV voltammograms were recorded using 5 mM $Ru(NH_3)_6^{2+/3+}$ in PBS 0.01M, from −0.60 V to +0.60 V at a scan rate of 50 mV/s. EIS was carried out using $Ru(NH_3)_6^{2+/3+}$ in PBS 0.01M, starting at open circuit potential (OCP) using a signal of amplitude 5 mV peak to peak in the frequency range of 10^9 Hz to 0.1 Hz. A Randles equivalent circuit was used to fit the obtained impedance spectra and to extract the values of the circuit component, which includes the charge transfer resistance (R_{Ct}), the electrolyte resistance ($R\Omega$), a constant phase element presenting the double layer capacitance (CPE), and the Warburg impedance (Zw). The Nyquist plot of EIS included a linear part at lower frequency range representing the diffusion-limited process and a semicircle portion at higher frequencies corresponding to the electron-transfer-limited process. The diameter of the semicircle of the Nyquist plot provides the magnitude of the R_{Ct}. The selection of the pair redox Ru $(NH_3)_6^{2+/3+}$ as probe (outer sphere model). was based on considering the different charges of the aptamer (positive) and the redox probe (negative) to achieve an optimal initial signal (without $A\beta O_{42}$) that allows a correct analysis of the modified electrode surface.

Square wave voltammetry (SWV) was used for the electrochemical sensing of the target molecules at a scan rate of 25 mV s^{-1} with a modulation amplitude of 50 mV and frequency of 25 Hz. The current is measured in the presence of 5 mM $Fe(CN)_6^{3-/4-}$ in 0.1 M KCl, PBS 0.01M; after depositing the resulting $MM_{GO\text{-}AuNPs}$-$Apt_{A\beta O42}$-$A\beta O_{42}$ on the working electrode surface of DRP-110 SPCE. The variation of the cathodic current is related to the concentration

of the target biomarker. In this case, we decided to use the $Fe(CN)_6^{3-/4-}$ probe, because is more stable than the pair redox $Ru(NH_3)_6^{2+/3+}$ and does not appear the problem described above.

In all the electrochemical sensing of the target molecules, the electrode must be in a horizontal position with a small magnet in the working electrode surface, so the MM are attached to it. The signals obtained were analyzed by the NOVA software predefined by Metrohm.

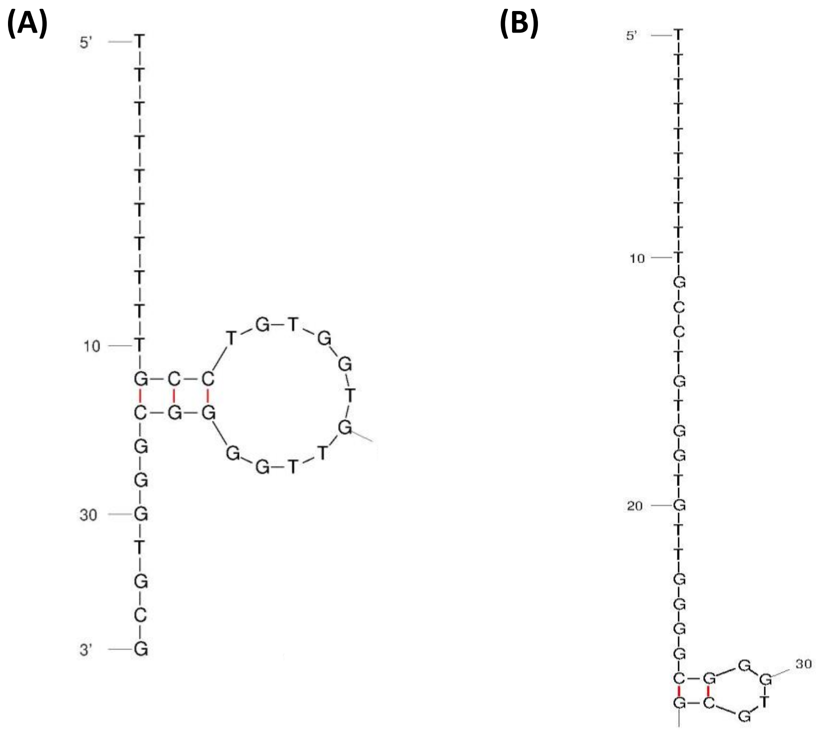

Figure S1. Possible folding of aptamer structure as calculated using Mfold Web server software.

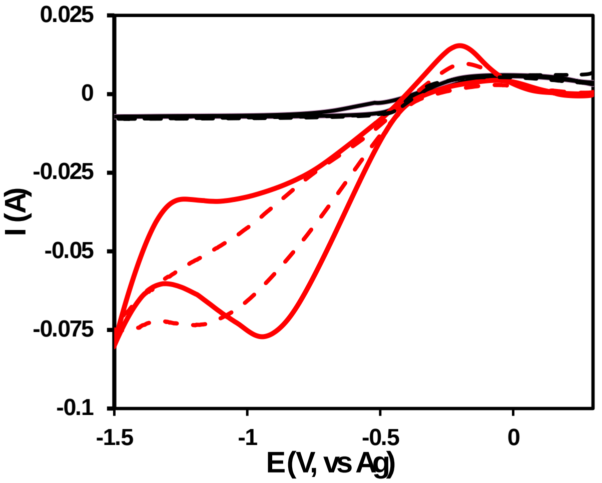

Figure S2.- First (----) and last after 10 cycles (____) of the cyclic voltammetry of the outer layer electrosynthesis of MM_{GO} (**black**) and $MM_{GO\text{-}AuNPs}$ (**red**).

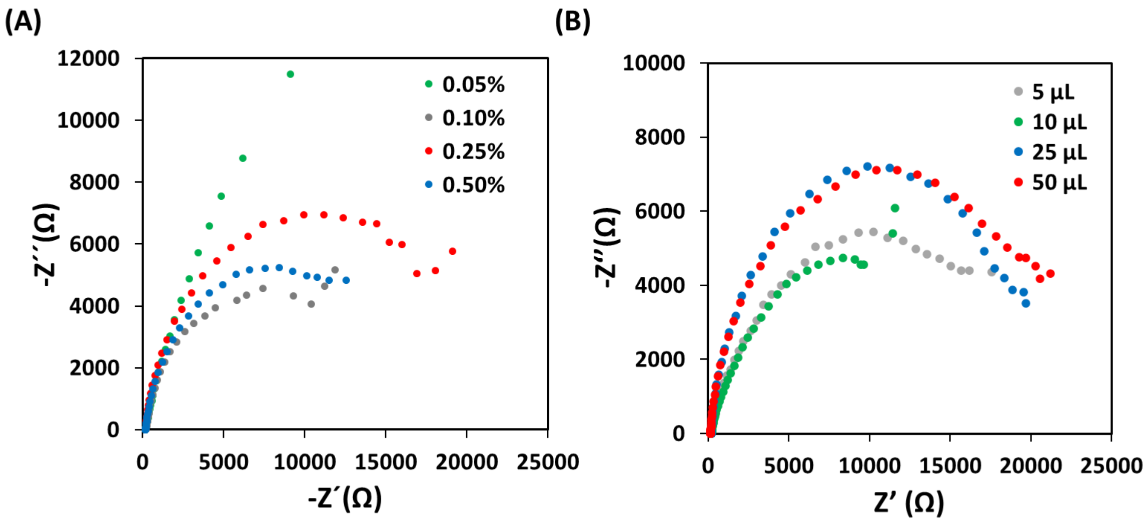

Figure S3.- Optimization of percentage of $HAuCl_4$ in the synthesis of out layer GO-AuNPs (A) and volume of MM (B). Experimental conditions: 10 [illegible]M of specific aptamer, a fixed excess of $A\beta O_{42}$ and 5 mM $Ru(NH_3)_6{}^{3+/2+}$ in PBS 0.01M

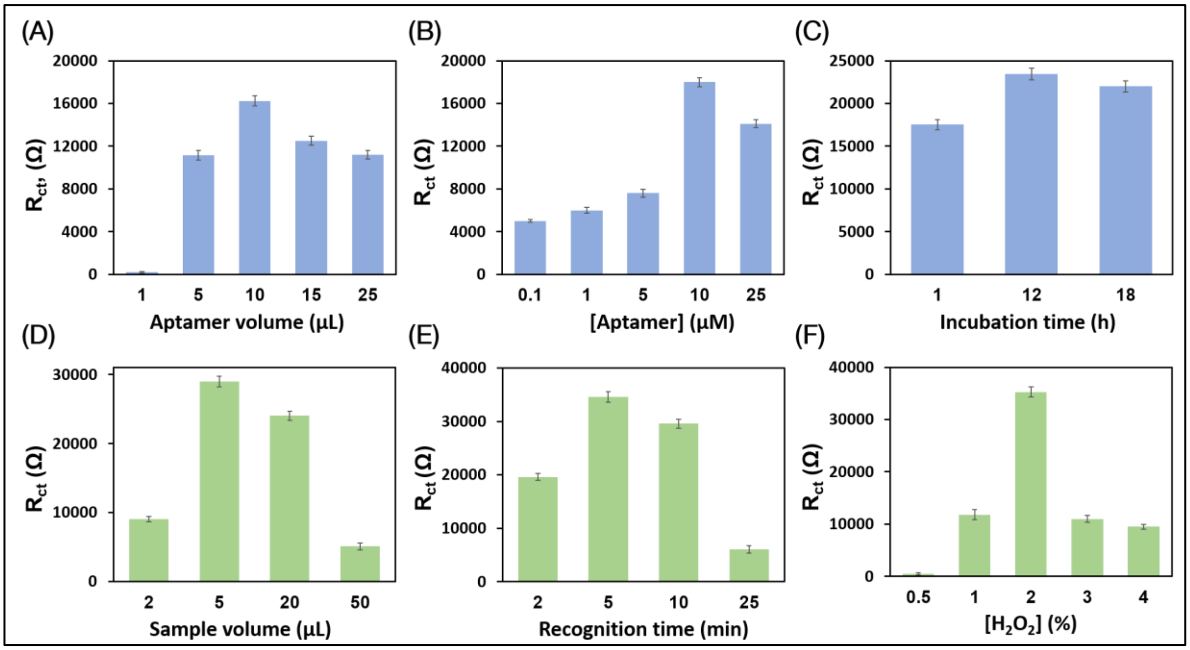

Figure S4.- Optimization of aptamer volume (A), aptamer concentration (B), aptamer incubation time (C), sample volume (D), sample recognition time € and hydrogen peroxide concentration (F). Conditions: 1 ng mL^{-1} of AβO$_{42}$ and 5 mM Ru$(NH_3)_6$ $^{3+/2+}$ in PBS 0.01 M.

Table S1. Overview of label free based-electrochemical bioassay for amyloid-β oligomers determination.

Electrode	Technique	Biorecognition element	LR (LOD)	Sample (volume)	Analysis time	Ref.
MCH Au-rod electrode	EIS	Aptamer	10^{-1} - 500 nM (30 pM)	Spiked artificial CSF	30 min	31
POPA coated Au electrode	EIS	PrPc	10^{-3}-10^{3} nM (0.5 pM)	Hamster ovary cell medium	20 min	45
PTAA-PEDOT-AuNPs-Au electrode	EIS	PrPc	10^{-8}-10^{4} nM (10^{-5} pM)	Mice tissue (2mL)	15 min	50
Gold nanostars electrode	EIS	PrPc	5 10^{-3}–0.2 nM (2 10^{-3} nM)		-	59
Ferrocene- MUA-MNH/Au electrode	SWV	Peptide	0.48-12 nM (0.24 nM)	Spiked fetal bovine serum	20 h 30 min	68
AuNPs-E-Ppy-3-COOH-Au electrode	EIS	PrPc	10^{-9}-10^{3} nM (10^{-6} pM)	Spiked cerebrospinal fluid and blood test of mice (10mL)	1h	69
Pt/Ti-SiO$_2$-APTS - IDC chip	Capacitive reactance	Aptamer	2.16 10^{-9} - 216.4 nM (0.02fM)	Real plasma (10 µL)	2h	70
Adamantine-PrP/AgNP	LSV	PrPc	10^{-2}–200 nM (6 pM)	Spiked CSF and serum (20 µL)	20 min	71

nanocomposites-Au electrode						
Poly(curcumin-Ni) electrode	EIS	Curcumin	10^{-3} - 5 nM (1 pM)	Artificial CSF (10 mL)	30 min	72
Au dendrite-PPy-3-COOH//PrPC-Au electrode	EIS	PrPc	10^{-9}-10 nM (1 aM)	Spiked Artificial CSF and blood (5mL)	-	73
$MM_{GOAuNPs}$-SCPE	SWV	Aptamer	10^{-4}–0.1 nM (0.02 pM)	Real brain tissue, CSF and plasma (5μL)	5 min	This work

Abreviations: APTS (3-aminopropyl) triethoxysilane); IDC (impedance to digital converter); LSV (linear sweep voltammetry); LR (linear range); MCH (6-Mercapto-1-hexanol); MNH (9-mercapto-1-nonanol); MUA (11-mercaptoundecanoic acid); POPA (polytyramine/3-(4-hydroxyphenyl) propionic acid); PPy (polypyrrole); PrPc (fragment of the cellular prion protein); PTAA (thiophene-3-acetic acid)

REFERENCES

(1) Zuker, M. Mfold web server for nucleic acid folding and hybridization prediction. *Nucleic Acids Res* **2003**, *31* (13), 3406-3415. DOI: 10.1093/nar/gkg595 From NLM Medline.
(2) Montero-Calle, A.; Lopez-Janeiro, A.; Mendes, M. L.; Perez-Hernandez, D.; Echevarria, I.; Ruz-Caracuel, I.; Heredia-Soto, V.; Mendiola, M.; Hardisson, D.; Argueso, P.; et al. In-depth quantitative proteomics analysis revealed C1GALT1 depletion in ECC-1 cells mimics an aggressive endometrial cancer phenotype observed in cancer patients with low C1GALT1 expression. *Cell Oncol (Dordr)* **2023**, *46* (3), 697-715. DOI: 10.1007/s13402-023-00778-w From NLM Medline.
